# Supplementary material for: Impact of Preparative Isolation of C-Glycosylflavones Derived from Dianthus superbus on In Vitro Glucose Metabolism
Source: Molecules. 2024 Jan 9;29(2):339. doi: 10.3390/molecules29020339 (PMC10820209; doi:10.3390/molecules29020339)

Impact of Preparative Isolation of C-Glycosylflavones Derived from *Dianthus superbus* on *In Vitro* Glucose Metabolism

Zikai Lin [1,†], Xiaowei Zhou [1,†], Chen Yuan [1,2], Yan Fang [1,2], Haozheng Zhou [1], Zhenhua Wang [1], Jun Dang [2,*] and Gang Li [1,*]

Table of Contents

Figure S1. High resolution mass spectrometry of 2''-O-rhamnosyllutonarin 3

Figure S2. ^{1}H NMR Spectrum (600 MHz) of 2''-O-rhamnosyllutonarin (in MeOH-d_4) 3

Figure S3. ^{13}C NMR Spectrum (151 MHz) of 2''-O-rhamnosyllutonarin (in MeOH-d_4) 4

Figure S4. The HSQC of 2''-O-rhamnosyllutonarin 4

Figure S5. The HMBC of 2''-O-rhamnosyllutonarin 5

Figure S6. The HHCOSY of 2''-O-rhamnosyllutonarin 5

Figure S7. Ultraviolet (UV) spectra of 2''-O-rhamnosyllutonarin 6

Figure S8. IR spectra of 2''-O-rhamnosyllutonarin 6

Figure S9. ESI mass spectrum of 6'''-O-Rhamnosyllutonarin 7

Figure S10. ^{1}H NMR Spectrum (600 MHz) of 6'''-O-Rhamnosyllutonarin (in MeOH-d_4) 7

Figure S11. ^{13}C NMR Spectrum (151 MHz) of 6'''-O-Rhamnosyllutonarin (in MeOH-d_4) 8

Figure S12. Ultraviolet (UV) spectra of 6'''-O-Rhamnosyllutonarin 8

Figure S13. IR spectra of 6'''-O-Rhamnosyllutonarin 9

Figure S1. High resolution mass spectrometry of 2''-O-rhamnosyllutonarin

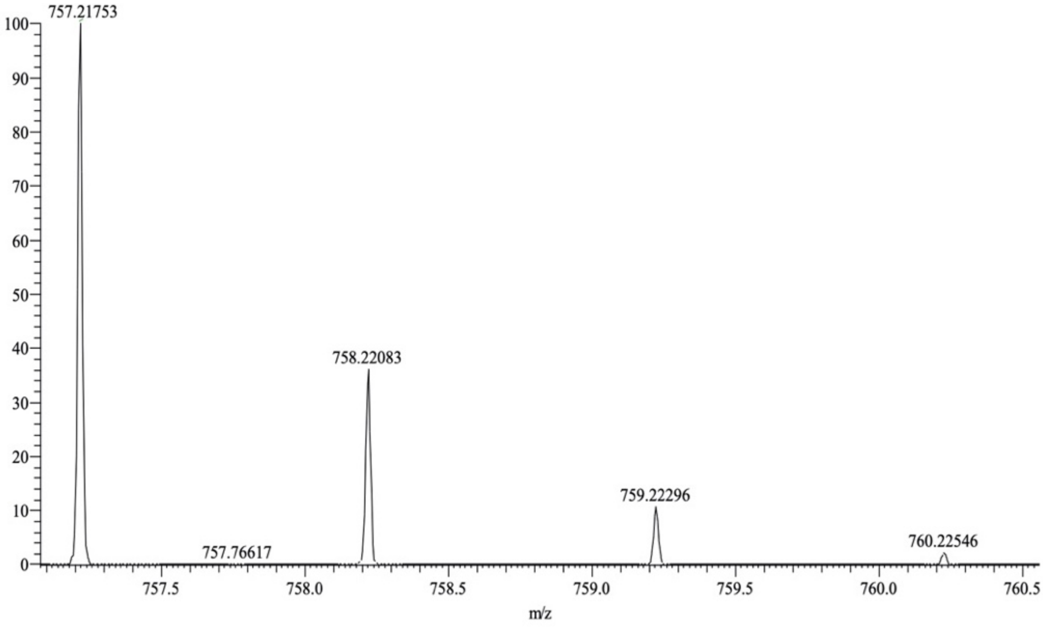

m/z	Theo. Mass	Delta (ppm)	RDB equiv.	Composition	
757.21753	757.21857	-1.37	13.5	C33 H41 O20	M+H

Figure S2. ^{1}H NMR Spectrum (600 MHz) of 2''-O-rhamnosyllutonarin (in MeOH-*d_4*)

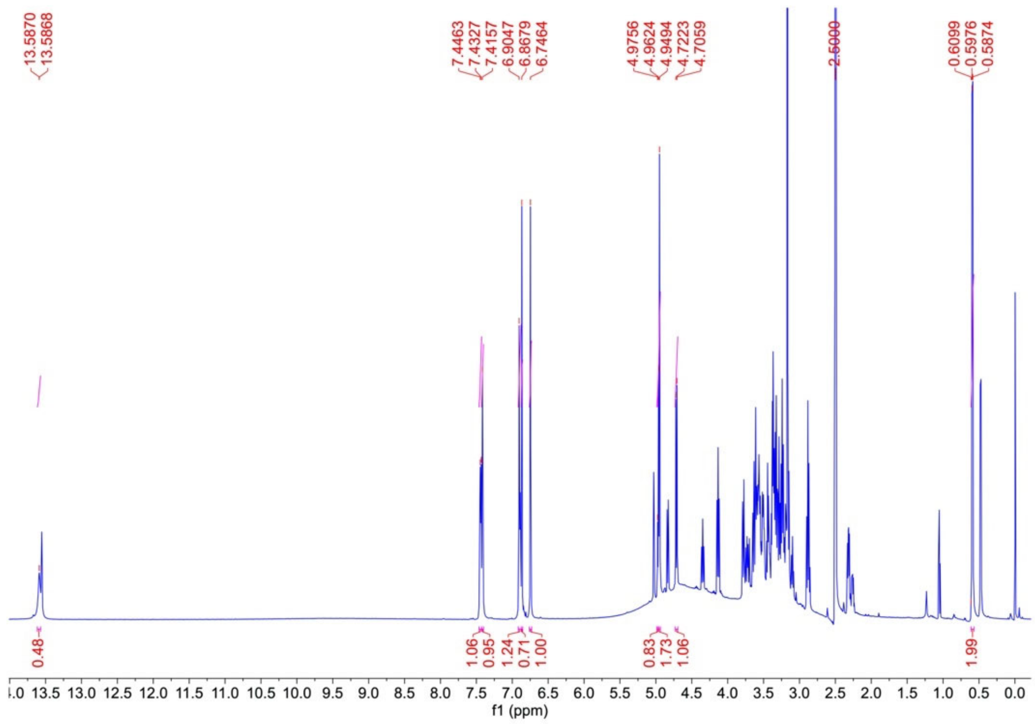

Figure S3. ^{13}C NMR Spectrum (151 MHz) of 2''-O-rhamnosyllutonarin (in MeOH-d_4)

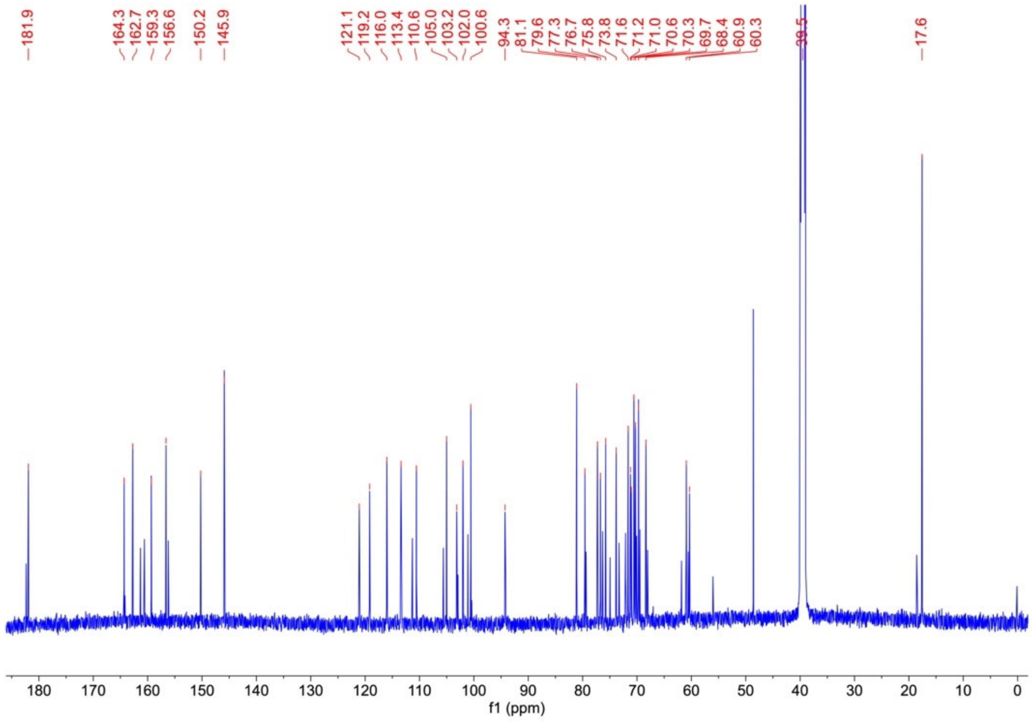

Figure S4. The HSQC of 2''-O-rhamnosyllutonarin

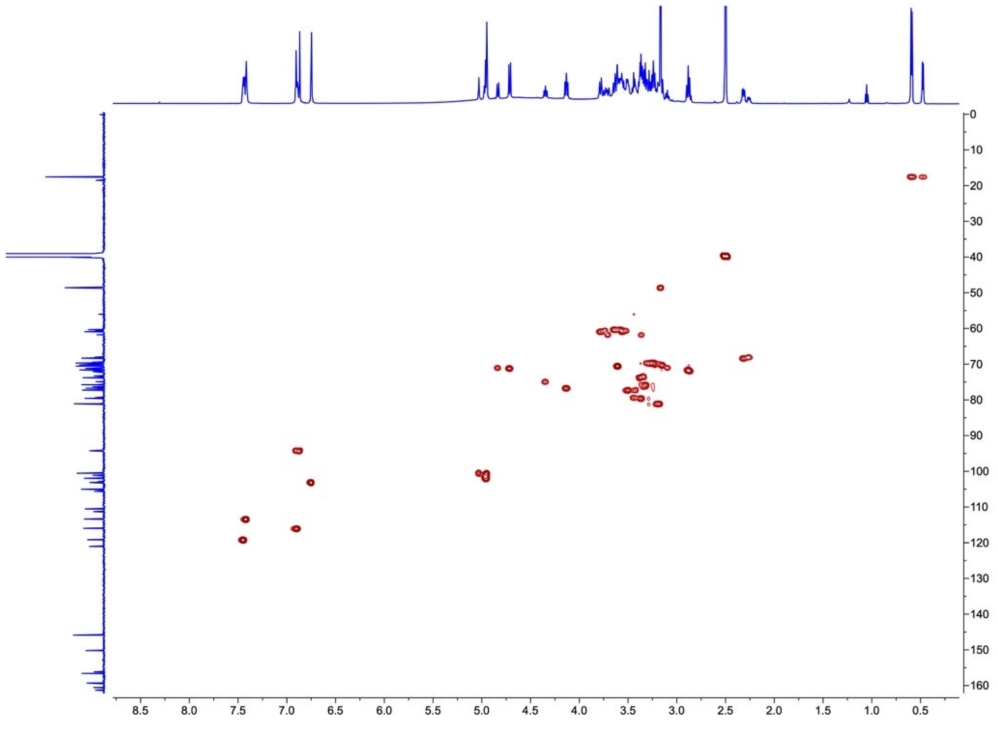

Figure S5. The HMBC of 2''-O-rhamnosyllutonarin

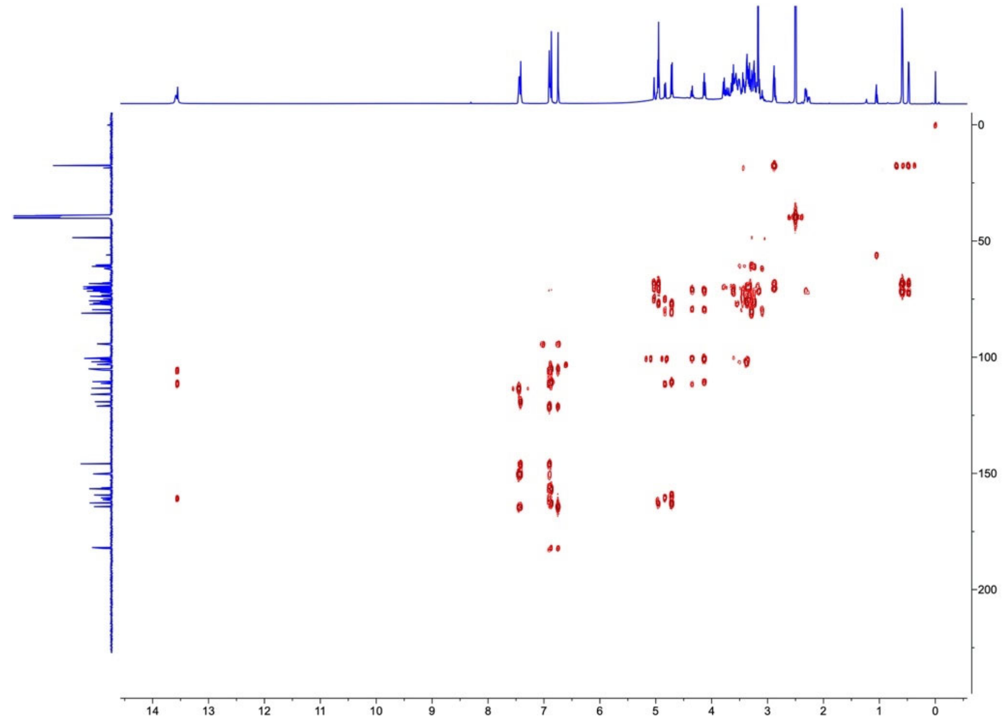

Figure S6. The HHCOSY of 2''-O-rhamnosyllutonarin

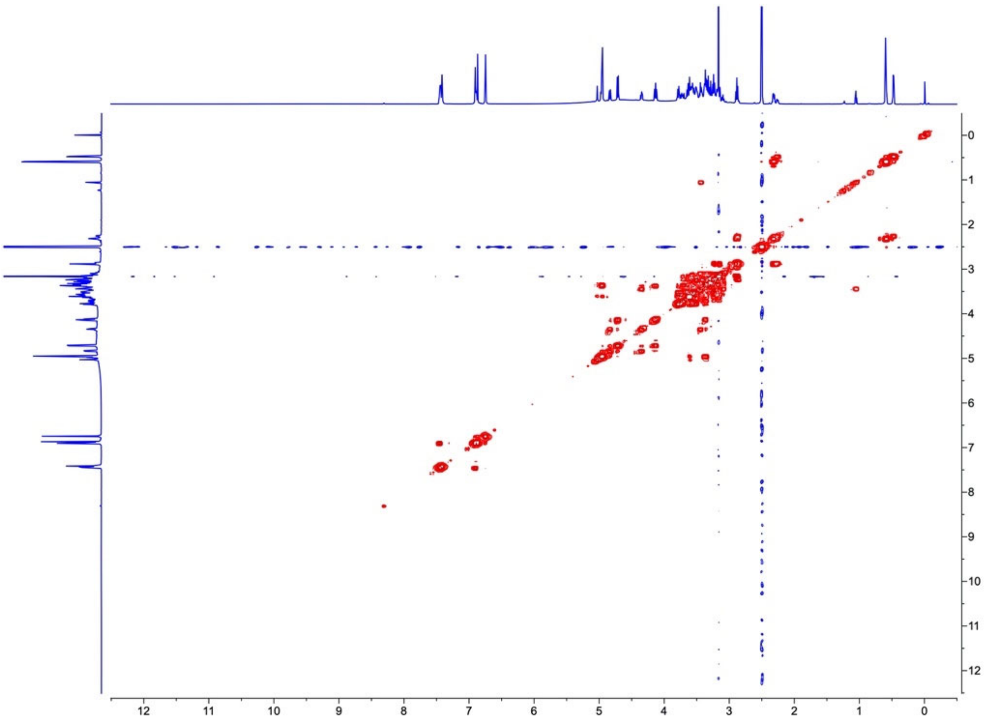

Figure S7. Ultraviolet (UV) spectra of 2''-O-rhamnosyllutonarin

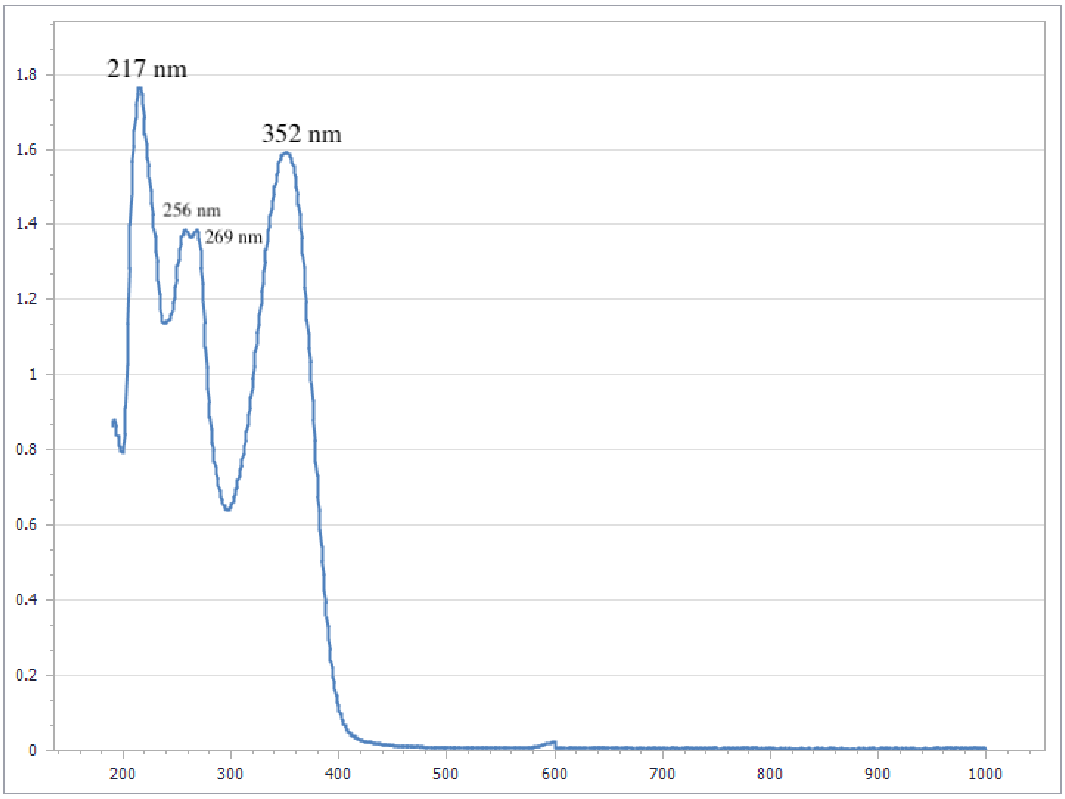

Figure S8. IR spectra of 2''-O-rhamnosyllutonarin

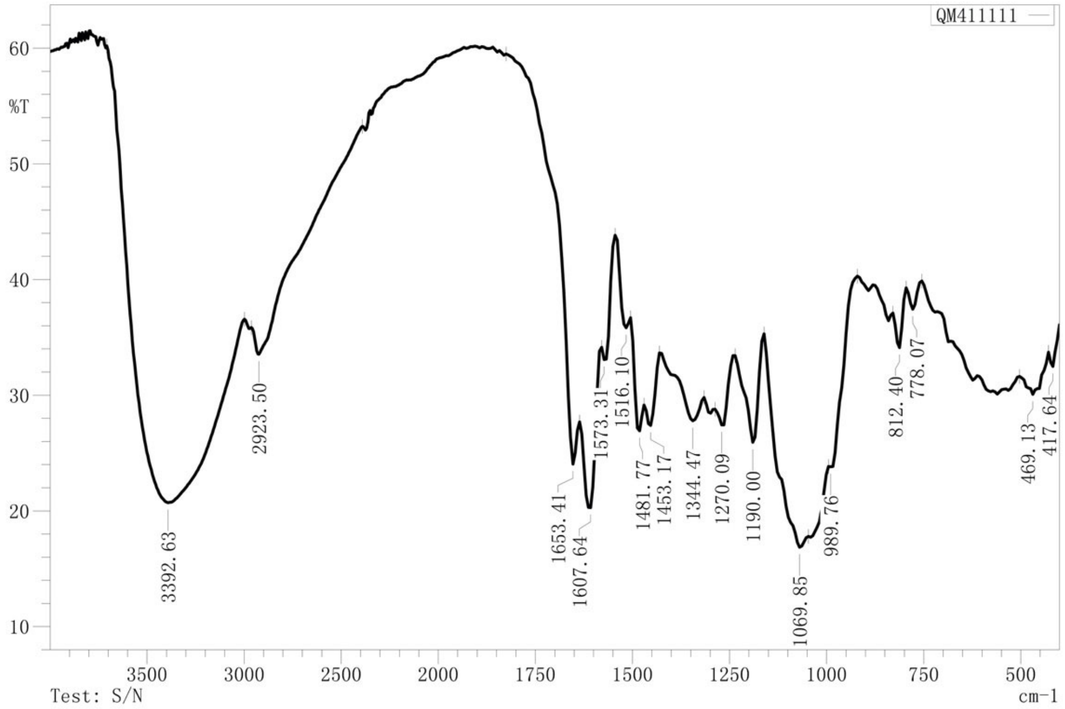

Figure S9. ESI mass spectrum of 6'''-O-Rhamnosyllutonarin

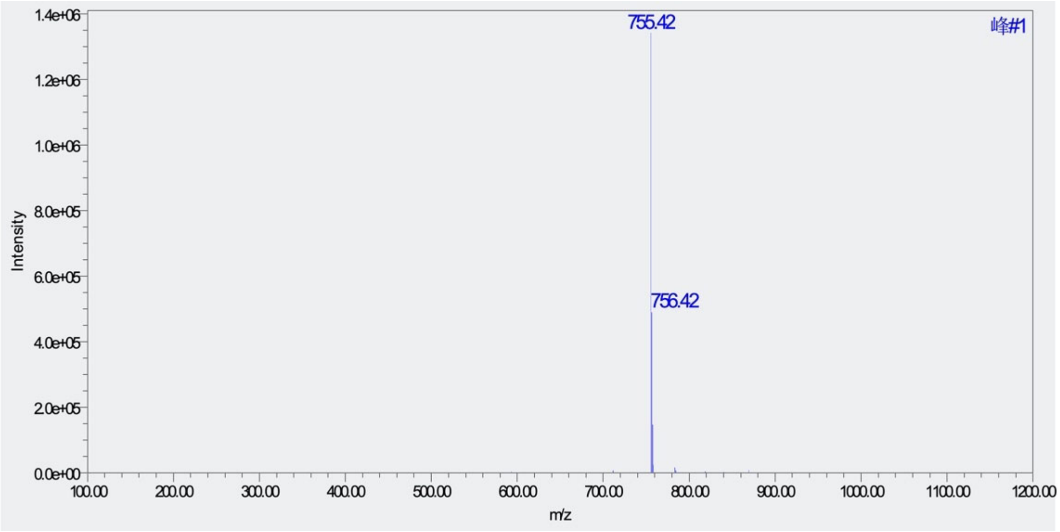

Figure S10. ^{1}H NMR Spectrum (600 MHz) of 6'''-O-Rhamnosyllutonarin (in MeOH-d_4)

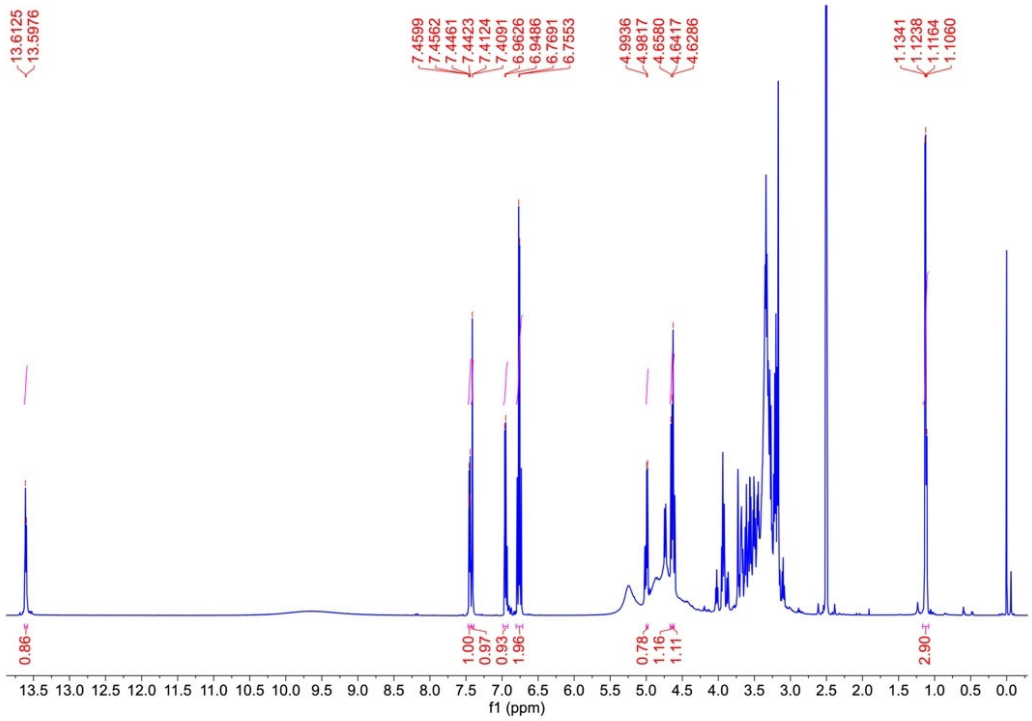

Figure S11. ^{13}C NMR Spectrum (151 MHz) of 6'''-O-Rhamnosyllutonarin (in MeOH-d_4)

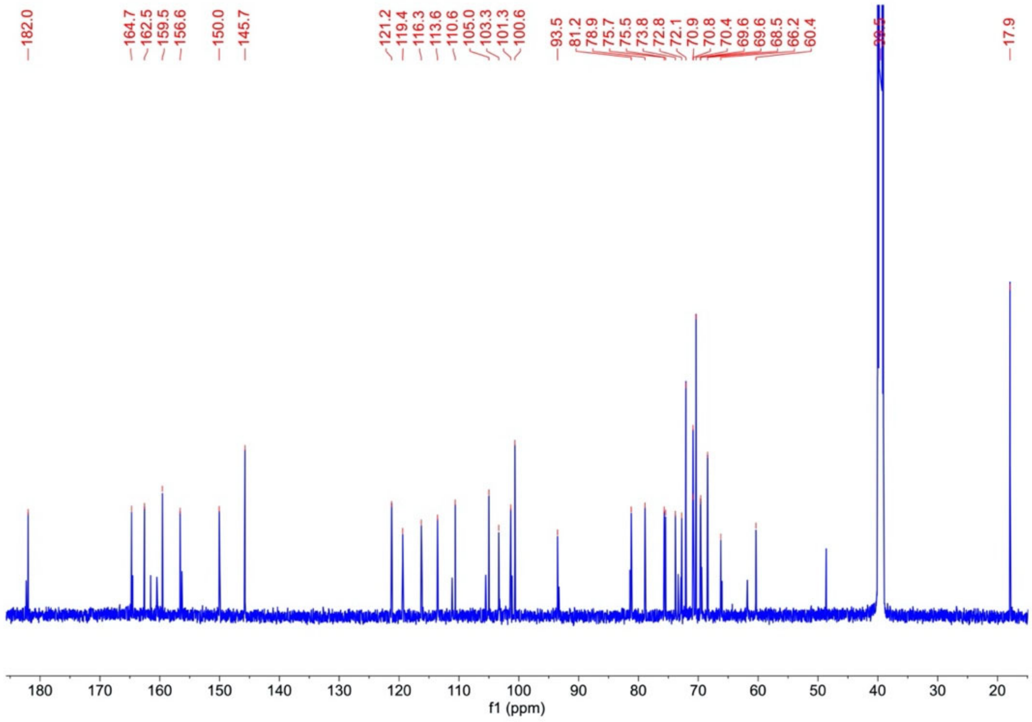

Figure S12. Ultraviolet (UV) spectra of 6'''-O-Rhamnosyllutonarin

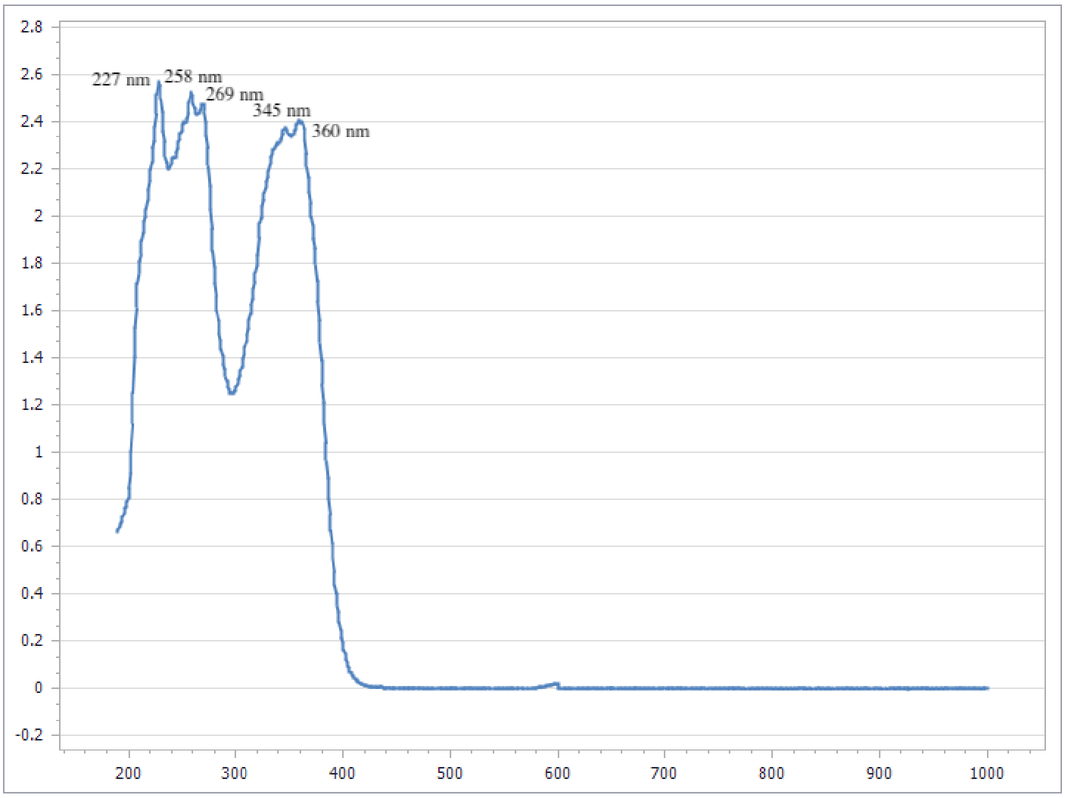

Figure S13. IR spectra of 6'''-O-Rhamnosyllutonarin

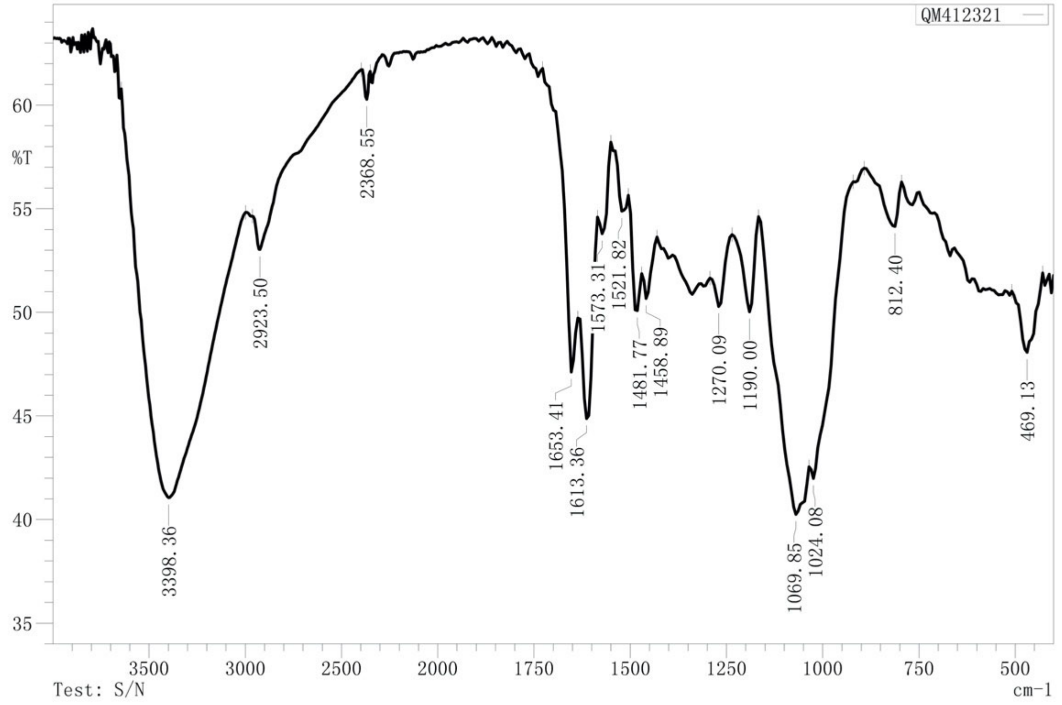

Supplement: Supplementary file 1 [file molecules-29-00339-s001.zip › molecules-2781111-supplementary.pdf]
